# Temporal Convolutional Neural Network-Based Prediction of Vascular Health in Elderly Women Using Photoplethysmography-Derived Pulse Wave during Exercise

**DOI:** 10.3390/s24134198

**Published:** 2024-06-28

**Authors:** Yue Xiao, Guixian Wang, Haojie Li

**Affiliations:** 1 Chinese Wushu Academy, Beijing Sport University, Beijing 100084, China; xiaoyue_scnu@163.com; 2 School of Physical Educantion and Sports, Sichuan Normal University, Chengdu 610101, China; 3 School of Physical Educantion and Sports, Beijing Normal University, Beijing 100875, China; 202121070037@mail.bnu.edu.cn

**Keywords:** temporal convolutional neural networks, pulse wave analysis, flow-mediated dilation, photoplethysmography, exercise

## Abstract

(1) Background: The objective of this study was to predict the vascular health status of elderly women during exercise using pulse wave data and Temporal Convolutional Neural Networks (TCN); (2) Methods: A total of 492 healthy elderly women aged 60–75 years were recruited for the study. The study utilized a cross-sectional design. Vascular endothelial function was assessed non-invasively using Flow-Mediated Dilation (FMD). Pulse wave characteristics were quantified using photoplethysmography (PPG) sensors, and motion-induced noise in the PPG signals was mitigated through the application of a recursive least squares (RLS) adaptive filtering algorithm. A fixed-load cycling exercise protocol was employed. A TCN was constructed to classify flow-mediated dilation (FMD) into “optimal”, “impaired”, and “at risk” levels; (3) Results: TCN achieved an average accuracy of 79.3%, 84.8%, and 83.2% in predicting FMD at the “optimal”, “impaired”, and “at risk” levels, respectively. The results of the analysis of variance (ANOVA) comparison demonstrated that the accuracy of the TCN in predicting FMD at the impaired and at-risk levels was significantly higher than that of Long Short-Term Memory (LSTM) networks and Random Forest algorithms; (4) Conclusions: The use of pulse wave data during exercise combined with the TCN for predicting the vascular health status of elderly women demonstrated high accuracy, particularly in predicting impaired and at-risk FMD levels. This indicates that the integration of exercise pulse wave data with TCN can serve as an effective tool for the assessment and monitoring of the vascular health of elderly women.

## 1. Introduction

Vascular health in the elderly is a growing concern. As we age, vascular health gradually deteriorates, leading to a range of vascular-related disorders such as hypertension [1], atherosclerosis [2], and decreased endothelial function [3]. These vascular health disorders not only affect the quality of life of older adults but also increase the risk of serious diseases such as cardiovascular disease [4].

Compared to men, older women are more likely to suffer from vascular health problems [5]. This may be closely related to factors such as women’s physiological characteristics, hormonal changes, and lifestyle [6,7]. A study by Virdis [8] found that the walls of blood vessels gradually become stiff and lose their elasticity as we age. In addition, fat and calcium may accumulate in the lining of blood vessels, which can lead to narrowing of the vessels and affect blood flow [9]. This condition is of particular concern in women because after menopause, the level of estrogen, which helps to maintain the elasticity and normal function of blood vessels, decreases in women [10]. A study by Watanabe [11] found that high blood pressure is a very common condition in older people, especially in elderly women because the decrease in estrogen affects the water–salt balance in the body and the tone of blood vessels. Hypertension increases the burden on the heart and blood vessels, and when prolonged, can lead to serious health problems such as heart disease, stroke, and kidney disease [12]. A study by Mikkola [13] found that atherosclerosis is the hardening and thinning of the walls of blood vessels due to the deposition of lipids and calcium in the vessels, which can increase the risk of heart disease and stroke. Older women may be more susceptible to atherosclerosis because of changes in hormone levels. Therefore, a deeper understanding of the influencing factors, predictive methods, and effective interventions for vascular health in older women is needed to safeguard the health of the female population.

As an important indicator of vascular health, pulse wave plays a key role in assessing cardiovascular function, vascular elasticity, and the degree of atherosclerosis. Pulse wave characteristics are closely related to the development of cardiovascular disease. Pulse wave is monitored by analyzing the propagation characteristics of the pressure wave generated by the heart as it pumps blood through the arteries [14,15]. This indicator reflects the elasticity of blood vessels and blood flow dynamics and is a very important physiological parameter for assessing an individual’s cardiovascular health [16]. Kerkhof [17] showed that normal pulse wave characteristics indicate good vascular elasticity and low cardiovascular risk, whereas abnormal pulse wave characteristics, such as increased pulse wave velocities, tend to signal atherosclerosis or other vascular pathologies. Further studies have also shown that changes in pulse waves can predict the risk of cardiovascular events. For example, Ji et al. [18] noted in their study that an increase in pulse wave velocity was significantly associated with an increase in future cardiac events and overall mortality. In addition, pulse waves can be used to assess the impact of therapeutic measures on vascular health, for example, in patients with hypertension, a reduction in pulse wave velocity due to antihypertensive treatment is often associated with a long-term reduction in cardiovascular risk [19,20]. Nonetheless, there are barriers to the current process of cardiovascular indicator collection, including slowness, high cost, and methodological complexity [21,22]. Addressing these challenges is important for the promotion of public health, particularly the prevention of cardiovascular disease and the improvement of overall health.

Temporal convolutional neural network (TCN), as an emerging neural network structure, has achieved remarkable results in the field of time series data analysis. It has the advantages of capturing long-term dependencies, efficient parallel computation, and parameter sharing, and has been widely used in speech recognition, natural language processing, and other fields [23,24]. Applying TCN to motion data analysis, combined with PPG technology, can effectively capture pulse wave characteristics during motion, thus realizing accurate prediction of vascular health. It can also assess vascular health more conveniently and accurately. While the vascular health of elderly women needs to be focused on, this study aims to combine neural network technology and exercise-related pulse waves to innovatively predict the vascular health of elderly women to provide a scientific basis and an effective means to promote the health of elderly women and to be able to more accurately, quickly, and conveniently predict the cardiovascular effects of exercise in elderly women.

## 2. Participants and Method

### 2.1. Participants

In this study, 492 elderly women aged 60–75 years were recruited based on criteria of good health, absence of significant medical conditions (e.g., heart failure, arrhythmias, uncontrolled hypertension), and no mobility or cognitive impairments, ensuring their capability for moderate-intensity exercise participation. Recruitment procedures adhered to the principles of the Declaration of Helsinki. Prior to participation, all prospective participants were provided with comprehensive information about the study’s risks and benefits and were required to provide written informed consent. The study protocol was approved by the Beijing Sport University’s Ethics Committee.

### 2.2. Method

#### 2.2.1. Study Design

**Figure 1:**
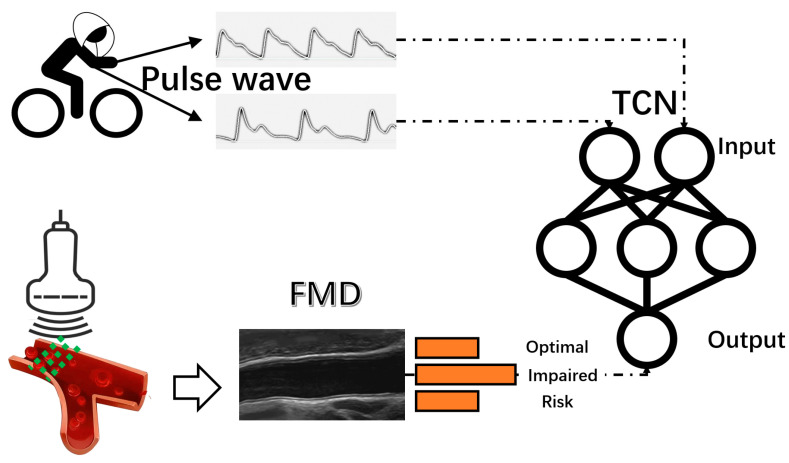
Overall experimental design. The overall architecture of the model: the input is the PPG data in exercise, the outputs are the three classifications of FMD—OPTIMAL, IMPAIRED, and RISK—and the TCN is used for training the model. Abbreviations: TCN: Temporal Convolutional Neural Network; FMD: flow-mediated dilation.

This study employed a cross-sectional design. Following the assessment of vascular health indicators, participants underwent a 30 min rest period before commencing the exercise protocol. Pulse wave data were collected 5 min before, during, and 5 min post-exercise. Testing occurred daily between the hours of 8:30 AM and 10:00 AM and between 3:00 PM and 5:00 PM (Figure 1).

#### 2.2.2. Vascular Indicators

In this study, vascular health indicators were determined using flow-mediated dilation (FMD), a non-invasive method that assesses endothelial function. Participants underwent FMD using a high-resolution Siemens Acuson S2000 ultrasound system (Munich, Germany) to obtain precise and dependable brachial artery images. The protocol began with a 5 min rest period in a controlled environment to establish baseline arterial dimensions. Subsequently, ischemia was induced by inflation of a blood pressure cuff (Omron Healthcare, Tokyo Japan) to 50 mmHg above the systolic pressure for 5 min. After cuff deflation, continuous ultrasound imaging was performed for 5 min, with the initial post-deflation minute being crucial for capturing peak dilation. Measurements were taken at end-diastole, with the R-wave on the electrocardiogram serving as the timing reference. FMD was quantified as the percentage increase in arterial diameter from the baseline to the post-ischemic values.

A three-level categorisation of FMD values was derived from a review of the literature, with values greater than 6% classified as ‘optimal’, reflecting a healthy endothelial function. FMD values between 6% and 5.2% were considered ‘impaired’, indicating some level of endothelial dysfunction. Finally, values below 5.2% were deemed ‘at risk’, suggesting a higher susceptibility to cardiovascular events [25].

In order to maintain methodological rigour, all scans were conducted by a single, well-trained sonographer who was unaware of the study’s broader aims. This approach ensured uniformity in data collection and minimized potential biases, thereby enhancing the reliability of the FMD outcomes.

#### 2.2.3. Pulse Wave Measurements

Pulse wave characteristics were non-invasively measured using photoplethysmography (PPG) sensors positioned on the fingertips and volar aspect of the forearm. This approach ensured signal accuracy and reduced interference from exercise-induced hand movements. PPG-based devices have been shown to detect pulsations in all skin types [26]. The fingertip PPG data were acquired with a PM-9000 device (Mindray Medical Systems, Beijing, China), optimized for precise pulse wave recording, while forearm PPG data were simultaneously captured using a HK-2000B wearable device (HUAKE Inc., Beijing, China). These devices were synchronized and calibrated to continuously record pulse wave velocity and amplitude during both rest and exercise, providing a detailed analysis of cardiovascular dynamics. Standardised protocols, including a 5 min acclimatisation period, were implemented to minimise variability and enhance the reliability of the PPG data, thereby accurately reflecting the participants’ cardiovascular responses to the exercise intervention. In this research, motion-induced noise in photoplethysmography (PPG) signals was addressed using a recursive least squares (RLS) adaptive filtering algorithm. The recursive least squares (RLS) algorithm was selected for its computational efficiency and its ability to dynamically update filter coefficients, which is ideal for real-time noise reduction in photoplethysmography (PPG) signals. Following the RLS preprocessing, a band-pass filter with a frequency range of 0.4–4 Hz was applied to the PPG signals. This frequency range preserves the physiologically relevant information essential for accurate cardiovascular parameter measurements.

#### 2.2.4. Exercise Protocol

The exercise intervention was conducted using a cycle ergometer (Lode Excalibur Sport, Groningen, The Netherlands), which was selected for its safety and adaptability for elderly women. The ergometer’s seat height was individually adjusted based on each participant’s stature to ensure optimal comfort and biomechanical alignment. The exercise load was set at a light intensity equivalent to 3 Newton meters (n.m), which is commonly used for warm-up purposes in this population. Participants were instructed to maintain a cadence of 60 revolutions per minute (rpm) throughout the six-minute duration of the exercise bout. This controlled cadence facilitated a steady state of light-to-moderate intensity exercise, which is beneficial for eliciting physiological responses without causing undue stress. During the cycling, participants were required to grip the handlebars, maintain an upright posture, and keep their gaze forward to ensure consistency in body position and to prevent any confounding effects of trunk movement on the pulse wave measurements. This standardized exercise protocol was designed to elicit a robust cardiovascular response while minimizing the risk of injury, making it suitable for the elderly female participants.

#### 2.2.5. Neural Network Construction

In this study, a carefully designed neural network was developed to handle time series data with the objective of accomplishing a Sequence-to-Sequence Classification task. Each sample’s input is a two-dimensional (2D) array composed of time and features. We have a total of 12 features, which include measurements from two different body sites, captured under two light sources—blue and green—and across three conditions: pre-exercise (5 min), during exercise (6 min), and post-exercise (5 min) for PPG data. The 2D arrays of all samples were combined to form a three-dimensional array with the shape of “samples-time-features”, which will serve as the input to the model. 

**Figure 2:**
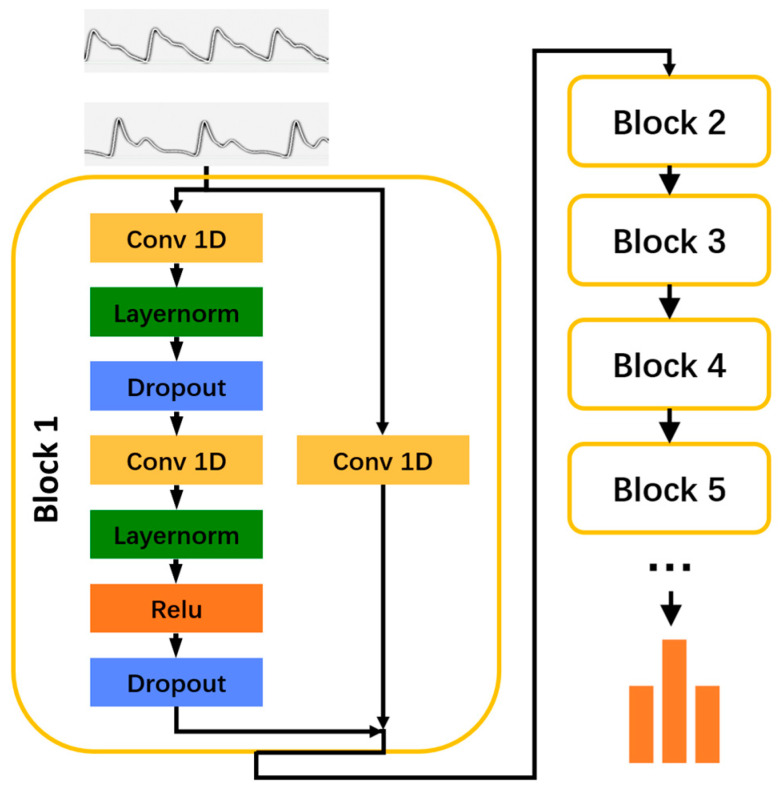
Structure of temporal convolutional neural networks. Abbreviations: Conv 1D: one-dimensional convolutional layer.

The network architecture comprised multiple identical blocks, each composed of a causal convolutional layer, a normalization layer, a dropout layer, and a ReLU (Rectified Linear Unit) activation layer [27]. This configuration was deliberately crafted to capture temporal dependencies in the pulse wave data. To enhance the network’s capacity to discern intricate temporal patterns and circumvent the vanishing gradient issue prevalent in deep learning, residual connections were deliberately incorporated between the blocks. The network culminated with a fully connected layer that integrated the extracted features to facilitate FMD classification. The hyperparameter optimisation was conducted using Optuna, a Python-based framework, which enabled the precise tuning of the number of blocks, neurons in the fully connected layer, dropout rate, and learning rate to align with the study’s objectives. Using Optuna, the optimal configuration for the model was determined to be 8 blocks, a fully connected layer with 64 neurons, and a dropout rate of 0.44. The model was trained using TensorFlow, a Python-based library for numerical computation, for 1000 epochs to ensure robust convergence and refine its predictive capabilities. The model’s robustness was further confirmed through a 3-fold cross-validation method, wherein the dataset was segmented into three parts, with two used for training and one for validation in each cycle. The trained model’s efficacy was quantitatively evaluated using a confusion matrix and accuracy metrics. Precision, indicative of the model’s accuracy in positive predictions, was used alongside accuracy to provide a detailed understanding of the model’s performance in classifying FMD levels. This focused assessment ensured a comprehensive evaluation of the model’s predictive accuracy and its effectiveness in discriminating between different FMD levels (Figure 2).

### 2.3. Statistical Analysis

The performance of the models was evaluated based on the accuracy rates obtained from each fold of the cross-validation process. The variability in these rates indicated the models’ consistency and reliability. For performance comparison, Long Short-Term Memory (LSTM) networks and Random Forest algorithms were additionally utilized to predict the outcomes. Subsequently, an Analysis of Variance (ANOVA) was conducted to evaluate the average accuracy rates across the folds for all three models. A *p*-value threshold of 0.05 was employed to ascertain statistical significance. All statistical analyses were conducted using Statistical Package for the Social Sciences (IBM Corp., Armonk, NY, USA) 24.0.

## 3. Results

**Figure 3:**
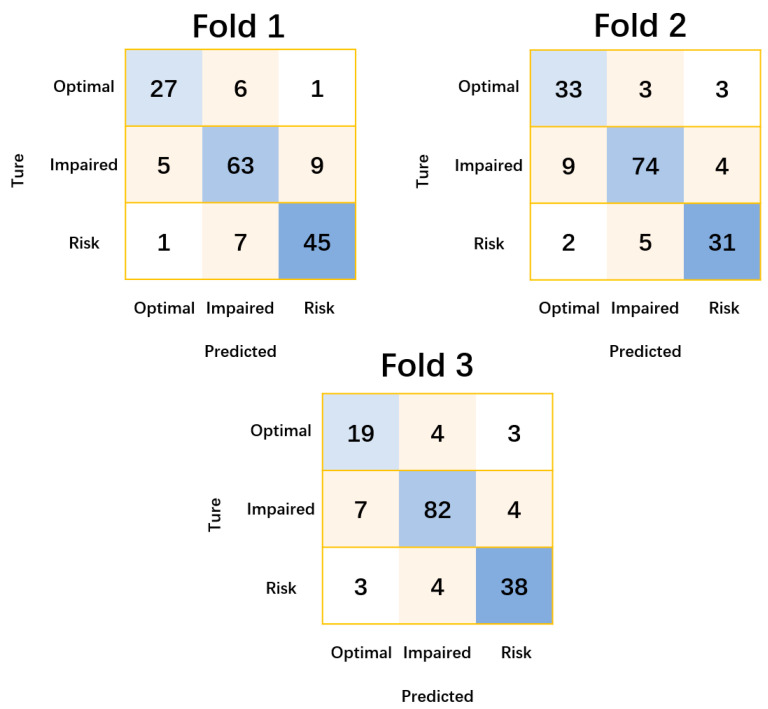
Confusion matrices for the prediction of flow-mediated dilation levels by temporal convolutional neural networks in each fold. Optimal, Impaired, and Risk are the three levels of blood flow-mediated dilatation, respectively, and the criteria for classification are referenced from epidemiological investigations [25]. The numeric values represent the number of specific categories, the higher the value, the darker the colour.

The results in Table 1 and Figure 3 show that TCN predicted FMD at the optimal level with an average accuracy of 79.3% and a coefficient of variation of 7%. TCN predicted FMD at the impaired level with an average accuracy of 84.8% and a coefficient of variation of 4.1%. TCN predicted FMD at the risk level with an average accuracy of 83.2% and a coefficient of variation of 2%.

The results in Table 2 show that the differences in accuracy between TCN, LSTM, and random forest in predicting FMD at the impaired level and risk level are significant. Multiple comparisons are significant in that TCN is significantly higher than random forest in predicting FMD at the impaired level and TCN is significantly higher than LSTM and random forest in predicting FMD at the risk level.

## 4. Discussion

The results of this study have important implications for cardiovascular health in older women. According to our findings, TCN achieved an average accuracy of 79.3%, 84.8%, and 83.2% in predicting different levels of vascular health. This suggests that the TCN model has a high predictive ability to effectively identify vascular health in older women. 

Older women face many challenges with cardiovascular health, such as atherosclerosis, hypertension, and cardiovascular disease. Timely and accurate assessment of vascular health is essential for the prevention and management of these diseases. Therefore, TCN, as an effective predictive tool, provides medical professionals with a convenient and reliable means to better understand the cardiovascular health status of older women. This finding not only facilitates early detection and intervention of potential cardiovascular health problems but also provides the basis for individualized medical care. For example, based on the predictive results of the TCN model, healthcare teams can develop targeted interventions, such as customized exercise programs, dietary recommendations, or medication regimens, to improve cardiovascular health in older women. 

Our results imply that the TCN model can efficiently identify vascular health in older women. Further results also suggest that exercise-related pulse wave characteristics have significant validity in predicting vascular health in older women. Consistent with Heffernan’s study [28], they found that normal pulse wave signatures usually indicate good vascular elasticity and low cardiovascular risk. In contrast, abnormal pulse wave characteristics, such as an increase in pulse wave velocity, tend to signal the possible presence of atherosclerosis or other vascular pathologies [29,30]. These findings not only provide us with a tool for accurate assessment of cardiovascular health in older women but also provide valuable guidance for future research and clinical practice. The combination of TCN-based modelling and pulse wave characterization may provide physicians with more accurate diagnostic and therapeutic recommendations to improve cardiovascular health in older women. Our findings also serve as a reminder of society’s concern for the health of the elderly population. By promoting a healthy lifestyle, including regular exercise, a balanced diet, and moderate rest, the risk of cardiovascular disease can be reduced, thereby prolonging the healthy lifespan of older women [31]. The promotion of such health promotion and preventive measures will have a positive impact on the health of the whole society [32].

In summary, the TCN-based neural network model can accurately predict the vascular health status of elderly women by analysing exercise-related pulse wave characteristics. This non-invasive assessment method is important for the health management of elderly women and can help to detect vascular health problems early so that appropriate interventions can be taken.

*Advantages*

(1) Non-invasive assessment method: the study uses exercise-related pulse wave features for assessment without invasive examination, which is more convenient and comfortable.
(2) High accuracy: By applying a neural network model based on pulse wave signatures, the study was able to accurately predict the vascular health status of elderly women, providing a reliable assessment tool for medical professionals.
(3) Personalized management recommendations: based on the prediction results, healthcare teams can develop individually tailored interventions, such as customized exercise plans, dietary recommendations, or medication regimens to improve cardiovascular health in older women.

*Limitations*

The results of this study are only applicable to the older female population, and applicability to other populations requires further research and validation. Overall, this study presents a non-invasive method for vascular health assessment by combining neural network technology and exercise data analysis and demonstrates its accuracy and potential in predicting vascular health in older women. This has important implications for health management and intervention in older women and provides new directions and possibilities for future research.

## 5. Conclusions

This study demonstrates a strong association between exercise-related pulse wave characteristics and vascular health and proves the feasibility and accuracy of a prediction method based on neural network modelling. The results of this study have important clinical implications. First, this non-invasive assessment method provides a convenient and comfortable tool for assessing vascular health in older women, avoiding the inconvenience and risks associated with traditional invasive examinations. Second, by accurately predicting vascular health, healthcare teams can identify potential cardiovascular risks early and develop personalized interventions to improve cardiovascular health in older women.

## Figures and Tables

**Table 1:** Accuracy of temporal convolutional neural networks in predicting different levels of blood flow-mediated dilatation.

FMD		Accuracy			
Levels	Fold 1	Fold 2	Fold 3	Average	CV
Optimal	79.4%	84.6%	73.1%	79.0%	7.3%
Impaired	81.2%	85.1%	88.2%	84.8%	4.1%
Risk	84.1%	81.2%	84.4%	83.2%	2.0%

Abbreviations: FMD: flow-mediated dilation; CV: coefficient of variation; Fold: 1-fold in 3-fold cross-validation. Optimal, Impaired, and Risk are the three levels of blood flow-mediated dilatation, respectively, and the criteria for classification are referenced from epidemiological investigations [25].

**Table 2:** Comparison of prediction accuracy between TCN and other models.

		FMD Levels	
	Optimal	Impaired	Risk
LSTM	67.8% ± 10.4%	71.5% ± 6.1%	65.4% ± 11.4%
Random Forest	65.8% ± 2.8%	60.7% ± 9.9%	67.2% ± 4.7%
TCN	79.0% ± 5.8%	84.8% ± 3.5% **^b^**	83.2% ± 1.7% **^a^^,^^b^**
*F*	3.06	8.96	5.62
*p*	0.121	0.016	0.042

Note: **^a^** indicates that the difference is significant when compared to LSTM. **^b^** indicates that the difference is significant when compared to Random Forest. F-values and *p*-values are the results of one-way ANOVA. F-values reflect overall group differences. Abbreviations: FMD: Flow-mediated dilation; LSTM: Long Short-Term Memory; TCN: Temporal Convolutional Neural Networks. F: F-statistic; *p*: *p*-value. Optimal, Impaired, and Risk are the three levels of blood flow-mediated dilatation, respectively, and the criteria for classification are referenced from epidemiological investigations [25].

## Data Availability

The data that support the findings of this study are available on request from the corresponding author.
